# Editorial

**Published:** 1864-04

**Authors:** 


### Editorial.

DEATH OF DR. O. L. LEONARD.—At the meeting of the Chicago Medical Society, on Friday, April 1st, the following resolutions were adopted:—

WHEREAS, in the order of Divine Providence, our friend and colleague, Dr. O. L. LEONARD, has been called from our midst.

*Resolved*, That, while deploring his death, as the loss of a Christian gentleman and a kind and faithful fellow laborer, we bow with humility before the will of Him who is alike infinite in wisdom and beneficence.

*Resolved*, That we recognized in our departed brother those honorable and noble qualities of mind and heart that endeared him alike to his family, his patients, and his confreres.

*Resolved*, That we hereby tender our heartfelt sympathies to the family of our deceased associate.

*Resolved*, That the Secretary of this Society be directed to present a copy of these Resolutions to the relatives of the deceased, and to request their publication in the medical journals and newspapers in this city.

E. MARGUERAT, M.D., *Secretary*.

CHICAGO MEDICAL SOCIETY.—The annual meeting of this Society was held in the Society's Room, in the Court House, on Friday evening, April 1, 1864. There was a full attendance of members, and the election of officers was conducted in a spirited and harmonious manner. The meetings of the Society have been better attended all the past year than at any previous time, and there are gratifying indications of increased activity and usefulness in the future. The following officers were chosen for the ensuing year:—

President, ....................M. O. HEYDOCK, M.D.
Vice-President, ............THOS. BEVAN, M.D.
Secretary and Treasurer, E. MARGUERAT, M.D.

Delegates to the State Medical Society. Drs. A. FISHER, J. BARTLETT, and IRA HATCH.

Delegates to the American Medical Association. Drs. C. G. SMITH, M. O. HEYDOCK, and A. FISHER.

The announcement of the Standing Committee on the Sanitary Condition of the City was deferred until the next meeting.

---

ANNUAL MEETING OF THE ILLINOIS STATE MEDICAL SOCIETY. The annual meeting of this Society will be held in the Common Council Room, in Chicago, commencing at 10 o'clock A.M., of Tuesday, May 5th.

The following is a list of the *Chairmen* of Committee who are expected to report at the said meeting. We hope none of them will report absent or unprepared:—

On Practical Medicine, Dr. N. S. Davis, of Chicago.
On Drugs and Medicines, Dr. F. R. Bailey, formerly of Joliet.
On Obstetrics, &c., Dr. DeLaskie Miller, of Chicago.
On Surgery, Dr. E. Andrews, of Chicago.
On Orthopœdic Surgery, Dr. Prince, of Jacksonville.
On the Delaterious Effects of the Use of Tobacco upon the Human System, &c., &c., Dr. N. S. Davis, of Chicago.

---

THE PEOPLE'S DENTAL JOURNAL.—The number of this valuable journal for April, comes to us with two new names added to the list of its editors. It is now edited by W. W. Allport, D.D.S., of Chicago; A. Hill, D.D.S., of Norwalk, Ct.; and J. Richardson, D.D.S., of Terre Haute, Ind.

The contents of the present number are interesting and valuable to all classes of readers.

---

ACKNOWLEDGMENT.—The Chicago Medical College having established a reading room and library, with the view of cultivating among its students a habit of familiarity with professional literature, a considerable number of the best medical journals of the country have been contributed by their conductors to the advancement of this object. I wish, in behalf of the reading room, to return them thanks for their liberality.

The following journals have been thus received:—

The American Medical Times,
The Boston Medical and Surgical Journal,
The Canada Lancet,
The Dental Cosmos,
The London Lancet,
The Medical and Surgical Reporter,
The Medical Examiner,
The Medical News and Library,
The People's Dental Journal,
The St. Louis Medical and Surgical Journal.

E. ANDREWS, M.D.,
*Librarian of Chicago Medical College.*

---

PHILADELPHIA, *March* 1, 1864.

*Dear Sir:*—The Transactions of the American Medical Association, Vol. XIV., are published, and now ready for delivery.

Should you desire a copy, please remit three dollars to my address.

As there are various methods by which the volume may be sent, inform me which you prefer. If by mail, please forward thirty-two cents in post-office stamps, that your postage may be prepaid. Very respectfully, CASPAR WISTER,
*Treasurer American Medical Association.*
No. 1303 Arch Street.

The following volumes are for sale:—

Proceedings of the Meeting of Organization, 50 cents.

(Vols. I., II., III., IV., and VI. are out of print.)

Vols. V., VII., VIII., and IX., *if taken collectively*, $5, for the set. If singly, $2 apiece.

Vols. X., XI., and XII. at $2 each.

Vols. XIII. and XIV. at $3 each.

### THE RELATIONS OF HEIGHT AND WEIGHT IN THE HUMAN BODY.

In the Statistical Society's Journal, of March last, a very interesting table is given, showing the growth of the human body from 18 up to 30 years of age, indicated by weight and height.

The averages were taken from upwards of 4,800 observations at all ages. Thus a lad of 18, if he be 5f. 4in. in height, speaking in round numbers, ought also to weigh somewhere about 8st. 10lbs. Given the age of 21, and the height 5ft. 5in., he should weigh 9st. 5lbs. Ascending still further, and assuming the age to be 25, and the height 5ft. 6in., the weight would be 10st. 5lbs., and at 30 years of age, with a height of 5ft. 6in., we ought to have the result 10st. 1lb. In fact so clear and demonstrable is this "law of increase in the growth of man," as determined by very extensive measurements taken at different times by scientific gentlemen, that we can almost work, as it were, in a rule of three sum, any one condition we like. Taking the converse of what we have already exhibited, we may say that if a lad of 19 weighs 9st. 4lbs., he ought to measure 5ft. 4in. and a little more; if at 22, 9st. 12lbs., he should be 5ft. 6in. in height, and so on.—*British Med. Journal, April* 25, 1863, *p*. 438.

USE AND ABUSE OF STIMULANTS IN FEVER—OCCASIONAL ANTIPHLOGISTIC TREATMENT.—A few days ago we saw at Guy's several patients convalescent from fever. In reference to them, Dr. Wilks remarked on the treatment of fever by stimulants. A young man, who had had typhus fever, and who had been covered with the ordinary mulberry rash, had recovered without any. As there appeared no need to give any, Dr. Wilks wished to prove to his class that alcohol was not always necessary in fever, and that he did not by any means consider alcohol as an antidote to fever, for he found the disease always ran its course under every form of treatment. He considered the rule laid down by many of the older physicians to be the correct one with regard to the treatment of all fevers; that in very many cases supervision was alone required, and that in others a stimulant plan was necessary; the only question being the quantity of alcohol required and the time when it was needed. He thought, therefore, that those who spoke of their success by the universal treatment by alcohol in all cases of fever, were adopting (to say the least) a very unscientific method, which was, in reality, one founded on such a reasoning as this: That severe cases of fever are benefited by alcohol, and mild ones are not killed by it, and, therefore, it is safer to give it to all. The same may be said of those who declare carbonate of ammonia to be *the remedy* for all cases of scarlatina. It is, no doubt, of great value in severe cases, and in mild ones it certainly will not kill the patient. Dr. Wilks would not say, however, that wine and spirits did no harm, for in some cases he believed they were decidedly injurious, especially in young persons with typhus fever and violent delirium. He had such a case under his care, in which he ordered cupping to the back of the neck, and which was followed by quiet and sleep. He was a total disbeliever in the change of type theory; for such a case as this, and two others which he had seen bled, and yet did well, entirely refuted such an opinion. Although he believed the present plan of treatment by support saved more lives, he was quite sure, that if no stimulants were given, and that if the patients were bled, the greater number would recover as heretofore.—*Med. Times & Gaz.*

---

TREATMENT OF DYSENTERY BY LARGE DOSES OF IPECACUANHA.—This plan of treatment was introduced, or brought prominently forward, by Dr. Docker, of Mauritius. The use of ipecacuanha in dysentery is by no means novel; but the employment of such large doses, and in the method described, is (Dr. Hillier said) of comparatively recent date. The plan is to give a drachm of tincture of opium, to apply a mustard plaster over the epigastrium, and, in twenty minutes, to give a drachm or a drachm and a half of powdered ipecacuanha in a very small quantity of peppermint water, or simple water. Sometimes half an ounce or an ounce of castor oil is given, with half a drachm of laudanum, before beginning the special treatment; this is however, usually found to be unnecessary. Vomiting is not often induced, and the cure is often immediate. A patient may be passing, every half hour or oftener, blood and mucus, or bloody serum with pus. They cease at once for about twenty-four hours; he then has a natural stool, and is well. The diet is farinaceous.

In May, 1862, Mr. Baylis, of Ceylon, wrote to Dr. Hillier that he had treated fifty or sixty cases in this way, and only lost three, who were in articulo mortis when they came under his care.

Dr. Hillier has had the opportunity of trying this mode of treatment at the Children's Hospital. It was in the case of a child, aged 4 years, suffering from sub-acute dysentery contracted in Barbadoes. He gave five minims of laudanum, and, in half an hour, fifteen grains of powdered ipecacuanha. There was no nausea or any unpleasant symptom caused by the medicine; and although the patient had previously passed five or six motions, containing much blood and mucus, every twenty-four hours, there was no evacuation for thirty-six hours. He then passed an ordinary feculent motion, and from that time he continued quite well. It is stated that ipecacuanha has the effect of rapidly healing large dysenteric ulcers. Dr. Hillier suggested that it might be worth while to try it in the diarrhœa dependent on tubercular ulceration, or in typhoid fever. The opium is supposed to act mainly in preventing vomiting, but it may, with ipecacuanha, have a more specific action on the disease.—*Ibid.*

---

Extraordinary Longevity.—Some rare instances of prolonged life have been lately presented in the obituary of *The Times*. In a recent number, the deaths of five gentlemen and three ladies are recorded, whose united ages amounted to 681, giving an average of upwards of 85 years to each. The eldest was a gentleman aged 102; the youngest, also of the same sex, was 80; the eldest lady was 87, and the youngest 82. Again, on the 19th inst., appears, amongst many others, ten whose united ages amounted to 862 years, the eldest being a lady aged 98, and the youngest, of the same sex, being 80 years of age; the eldest gentleman was 91, and the youngest 82. The same paper also gives a short notice of a lady who lived in a state of celibacy until the age of 60, when she married, and lived 47 years afterwards, having reached the extraordinary age of 107 years. On adding this lady's age to the 862, we have a grand total of 960, or an average of 88 years for each person. The following day (the 20th inst.) there appeared also eight ladies and one gentleman representing a total of 789 years—the fair sex, as usual, taking the lead, the eldest having reached 98 years, and the youngest 82; the gentleman was 83. The average age of this group would be 87 years and 8 months. *The Times* of the 21st, announced, amongst others, eight persons whose united ages amounted to 675, giving an average of 84 years and 4 months to each individual; the eldest was 93 and the youngest 80 years of age. Some curious and interesting mortality statistics could be compiled from the obituary of *The Times*.

---

M. Velpeau at the Academy of Sciences of Paris.—This eminent surgeon has just vacated the President's chair of this

Academy, and it redounds in no small degree to the honor of the profession that such an eminent post has been held and so worthily occupied by M. Velpeau. It reminds us of Sir B. Brodie presiding at the Royal Society. Our readers are aware that the Academy consists of numerous sections, the members of which are eminent in the different branches of science. Among these branches there is of course one for Medicine and Surgery, and the attainment of a membership in this section is the highest point towards which the imagination of French physicians and surgeons is wont to soar. M. Velpeau delivered, before leaving the chair, a most appropriate and touching speech, in which he especially dwelt on the fact of having started from the humble ranks of life, and having at last obtained the envied honor of presiding, for one year, over the Academy of Sciences of Paris.

---

The British Pharmacopœia.—In forming an opinion of the merits of this work, it is impossible to forget that the book has now been more than four years in preparation, and that it has cost a sum variously stated at £6000 or £8000. We have no interest in depreciating the value of literary and laboratory work, but we have no hesitation in asserting that, the work could have been as well prepared in less than twelve months by any moderately accomplished chemist and pharmaceutist, who would have thought himself richly rewarded with £400 or £500. No doubt the contentions between the three colleges about their rival preparations were frequent and severe; no doubt, the debates on such questions, as whether iodine should be called iodum or iodinium, and whether pil. galbani co. should, for the future, be known as pil. assafœtidæ co. or not, were long and fierce. We, however, have only to judge of the results. Although the book has been all these years in preparation, it bears evident marks of having been put together in a hurry. Some of the directions are incomplete and faulty. The bulk of the work is mere compilation. Much of it is taken up with prescriptions such as medical men should be left to write at their discretion; *e. g.*, mist. creasoti, mist. scammoni, pil. plumbi c. opio. The chemistry does not fairly represent the science of the day. Taken altogether, the book, as the production of the collective wisdom of the Medical Council, is far from likely to increase the respect which that learned body has inspired.—*Chemical News.*

---

Infantile Mortality in New York City.—From the elaborate statistical tables prepared by Dr. Cyrus Ramsay, Registrar of Records and Statistics for the City of New York, we learn that a steady improvement has been going on in that city for the past ten years, in regard to the mortality of children. This is shown in the fact, that the number of deaths of children each year, since 1851, has remained nearly the same or even smaller (except during the cholera year, 1854), while the population has rapidly increased, and during the time mentioned has actually doubled. The number of deaths of children under one year, as recorded by Dr. R., was as follows:—In 1851, 6891; in 1852, 6351; in 1853, 6661; in 1854, 7551; in 1855, 6771; in 1856, 6437; in 1857, 6405; in 1858, 7109; in 1859, 6599; in 1860, 6087; in 1861, 6189; in 1862, 5720; in 1863, 6118. The number of deaths of adults recorded in 1851, was 7775; and in 1863, 10,596. The deaths of adults and children of all ages was, in 1851, 21,748; and in 1863, 25,196.

---

Action for Alleged Illegal Detention in an Asylum.—An action for damages for "wrongous and illegal detention," in Saughton Hall Lunatic Asylum, has been occupying the attention of the Scotch Courts. The defenders in the action are Drs. Smith and Lowe, owners of the asylum, in which Angus Macintosh, Esq., was confined for about six weeks, in the summer of 1852, under a warrant of one of the sheriff-substitutes of Edinburgh, granted on a certificate of two doctors, that he was of unsound mind. Two or three years ago, he had another action of damages against these doctors, who signed the certificate, and in that action he was unsuccessful, the jury finding, in point of fact, that he was insane when the certificate was granted. He tried to obtain a new trial and failed, and has brought this action to have the privilege of telling his own story to the jury. And he did tell it very well, and was a most amusing and clever witness, giving his evidence in a frank, nonchalant, gentlemanly way, expressing himself elegantly, and dexterously avoiding awkward admissions. He remembered all his mad-like pranks, had reasons for them all, and ascribed all his extravagances to a three months' spree in London. After a lengthy and able summing up by the judge, the jury, after an absence of three hours, returned a verdict for the defendants, by a majority of nine to three.—*British Medical Journal.*

